# Availability of essential medicines for non-communicable diseases: a scoping review of challenges and opportunities

**DOI:** 10.1136/bmjgh-2025-019634

**Published:** 2025-11-27

**Authors:** Iris R Joosse, Aukje Mantel-Teeuwisse, Hendrika A van den Ham, Lourdes Cantarero Arevalo

**Affiliations:** 1 Utrecht WHO Collaborating Centre for Pharmaceutical Policy and Regulation, Division of Pharmacoepidemiology and Clinical Pharmacology, Utrecht Institute for Pharmaceutical Sciences (UIPS), Utrecht University, Utrecht, The Netherlands; 2 WHO Collaborating Center for Research and Training in the Patient Perspective on Medicines Use, Department of Pharmacy, Faculty of health and Medical Sciences, University of Copenhagen, Copenhagen, Denmark

**Keywords:** Health Services Accessibility, Diabetes, Cardiovascular disease, Cancer, Pharmacology

## Abstract

**Introduction:**

Given the critical role of medicines in reducing the burden of non-communicable diseases (NCDs), we analysed factors that hinder the availability of quality medicines for managing NCDs at the point of service delivery, with a particular focus on low-income and middle-income countries.

**Methods:**

In this scoping review, literature published in PubMed, Embase, Web of Science, the World Bank eLibrary and the WHO’s Institutional Repository for Information Sharing (WHO IRIS) was collected (January 2009–May 2025). Literature was excluded if it did not specifically address NCD medicines, discussed global rather than national determinants of availability only, failed to detail supply chain inefficiencies or exclusively reported on other dimensions of access (ie, pricing/affordability, trial access, regulatory access). From cross-sectional assessments of medicine availability, mean availability was extracted, and from quantitative, interventional studies, details on the intervention and its effectiveness on NCD medicine availability were extracted. From the remaining studies, descriptions of barriers and recommendations or empirically proven interventions to improve availability were extracted and mapped according to the pharmaceutical value chain (PVC).

**Results:**

Our review of 3348 records identified 83 eligible studies. The mean availability of NCD medicines reported in cross-sectional surveys was suboptimal (<80%) in 39 of 46 surveyed countries. We found barriers to available NCD medicines across all components of the PVC. Deficiencies in governance, financing, the health workforce, health information systems and service delivery underscore the interlinkage of various health system building blocks and stakeholders in determining availability. Nonetheless, most barriers pertained to supply chain inefficiencies, followed by challenges in regulatory systems and quality monitoring.

**Conclusions:**

The evidence highlights the need for political commitment to NCDs, broad stakeholder involvement, integrated stock management systems and increased human resources to make NCD medicines more available to patients worldwide.

**WHAT IS ALREADY KNOWN ON THIS TOPIC**

- Availability of medicines at the point of service delivery is a key aspect of access and non-communicable disease (NCD) management.
- Estimates of global availability of NCD medicines are outdated, and clarity on factors that contribute to shortages or limited access is lacking.

**WHAT THIS STUDY ADDS**

- The updated evidence shows that NCD medicine availability has not (consistently) improved since the start of this century, often remaining well below the aspired target of 80% availability.
- Supply chain inefficiencies are important contributors to unavailable NCD medicines, as are deficiencies in governance, financing, the health workforce, health information systems and service delivery.

**HOW THIS STUDY MIGHT AFFECT RESEARCH, PRACTICE OR POLICY**

- The evidence underscores the role and interlinkage of different processes, stakeholders and health system building blocks—other than those in the supply chain only.
- By applying a health systems lens to understanding barriers and facilitators to availability, challenges to and opportunities for improved availability of NCD medicines were identified across the pharmaceutical value chain.
- This study highlights the often superficial understanding of factors determining availability at the point of medicine delivery, calling for in-depth exploration of these factors by researchers in this field.

## Introduction

 On top of the unfinished agenda for infectious diseases in low-income and middle-income countries (LMICs), non-communicable diseases (NCDs) constitute a growing proportion of morbidity and mortality worldwide.^1 2^ An estimated 71% of all deaths is contributed to NCDs; 86% of these premature deaths occur in LMICs.^3^ Major causes of mortality are cardiovascular diseases (CVDs), cancers, chronic respiratory diseases and diabetes, together accounting for over 80% of all premature NCD deaths.^3 4^ Besides the four major therapeutic areas, mental disorders are a leading cause of disability globally.^5^

In order to lower the burden of NCDs, a global action plan (GAP) for the prevention and control of NCDs was agreed on in 2013.^6 7^ With surveys indicating between 12% and 18% lower availability of essential NCD medicines compared with medicines for acute conditions,^8^ improved access to essential medicines for preventing and treating NCDs emerged as a key priority within this action plan.^7^ Similar targets were later also integrated into the Sustainable Development Goals, with target 3.4 aiming to reduce premature mortality from NCDs by one-third, and targets 3.8 and 3.b aiming to achieve universal health coverage, including access to quality essential medicines for all by 2023.^9^

Despite the GAP setting a target of at least 80% availability of affordable essential medicines for NCDs,^7^ a 2019 global survey assessing national capacity for NCD health services revealed that only 10% of low-income countries had a set of 11 essential NCD medicines generally available, compared with 93% of the high-income countries.^10^ A systematic review seeking to assess public sector capacity to prevent and control NCDs likewise revealed critical gaps in essential medicines availability in LMICs.^11^ Given the critical role of medicines in reducing the burden of NCDs,^12^ improved access to essential NCD medicines thus remained a priority in the 2023–2030 extension of the GAP.^13 14^

Access to medicines is considered a multidimensional concept that includes affordability, availability, accessibility, acceptability and quality as a cross-cutting domain.^15^ Although availability stands out as a critical determinant of access, clarity on the root causes of unavailable NCD medicines is lacking. This scoping review aimed to analyse and document the factors that hinder the availability of quality medicines for managing NCDs at the point of service delivery at a country level. By focusing specifically on availability, we sought to identify critical challenges and barriers within healthcare systems that impede timely access to essential NCD medicines in LMIC. Additionally, we aimed to explore potential entry points within national decision-making processes and (policy) interventions that could enhance the availability of NCD medicines.

## Methods

This scoping review collected and reviewed evidence on NCD medicine availability reported in scientific articles and grey literature. To this end, the following key databases were searched by authors IRJ and LCA on 31 May 2025: PubMed, Embase, Web of Science, World Bank eLibrary/Open Knowledge Repository and the WHO’s Institutional Repository for Information Sharing (WHO IRIS). To identify relevant records, the search string contained three core elements: (1) medicines, (2) NCDs, including major therapeutic areas such as CVDs, diabetes, cancer, respiratory diseases and mental health and neurological conditions and (3) availability (see online supplemental annex 1). The reference lists of scoping and systematic reviews identified through this search were screened for additional relevant records by author IRJ through a snowball approach. We searched for (grey) literature published up to 15 years ago (January 2009 to May 2025).

Inclusion criteria were as follows: original research articles, working papers or reports providing in-depth information on physical availability (including factors determining availability) of NCD medicines. Factors associated with the physical availability of quality medicines included shortages or stockouts, supply chains and distribution, and substandard and falsified medicines. Eligible NCDs included diabetes, CVD, respiratory diseases such as asthma and COPD, cancer, including cancer pain, mental health and neurological conditions, including epilepsy. Full-text articles in English, French or Spanish were included. Screening and selection were conducted by IRJ or LCA, with any doubts being discussed between the researchers and, if necessary, with author AM-T.

Editorials, commentaries, letters, news articles and conference abstracts were excluded. Articles not reporting specifically on NCD medicines were excluded, as were articles exclusively reporting on access to medicines from the perspective of affordability (ie, prices, out-of-pocket expenditures, reimbursement coverage, pricing interventions), trial access (including compassionate access) or regulatory access (including accelerated or early access, time to registration and market access). Articles reporting on access to NCD medicines and availability at the patient or household level rather than at the point of service delivery were not eligible. Articles exclusively discussing global determinants of availability rather than national determinants were excluded, as well as articles failing to provide details on which supply chain inefficiencies occurred. Finally, cross-sectional studies of medicine availability were only included if availability was reported as a national/regional mean across one or multiple therapeutic groups, sectors (ie, public, private or other) or facility types (ie, pharmacy, hospital). Those studies reporting availability outcomes for individual medicines only without reporting group means were excluded.

Several types of data were extracted from eligible records by IRJ or LCA: (1) from studies presenting findings from cross-sectional assessments of physical availability at the point of service delivery, mean availability across sectors and facility types was extracted, and the main text was screened for any explanations for suboptimal availability; (2) from studies quantitatively evaluating the impact of an intervention intended to improve the availability of NCD medicines, details on the intervention and its effectiveness were extracted and (3) from the remaining studies, descriptions of barriers to physical availability at the point of service delivery were extracted, as well as any recommendations or empirically proven interventions to improve availability. Barriers and recommendations were mapped and categorised according to the Pharmaceutical Value Chain (PVC) as proposed by Joosse *et al*,^16^ with the addition of core component manufacturing. In contrast, pricing and reimbursement were disregarded, given the review’s focus on availability.

### Patient and public involvement

There was no patient or public involvement in the design or conduct of this study.

## Results

**Figure 1:**
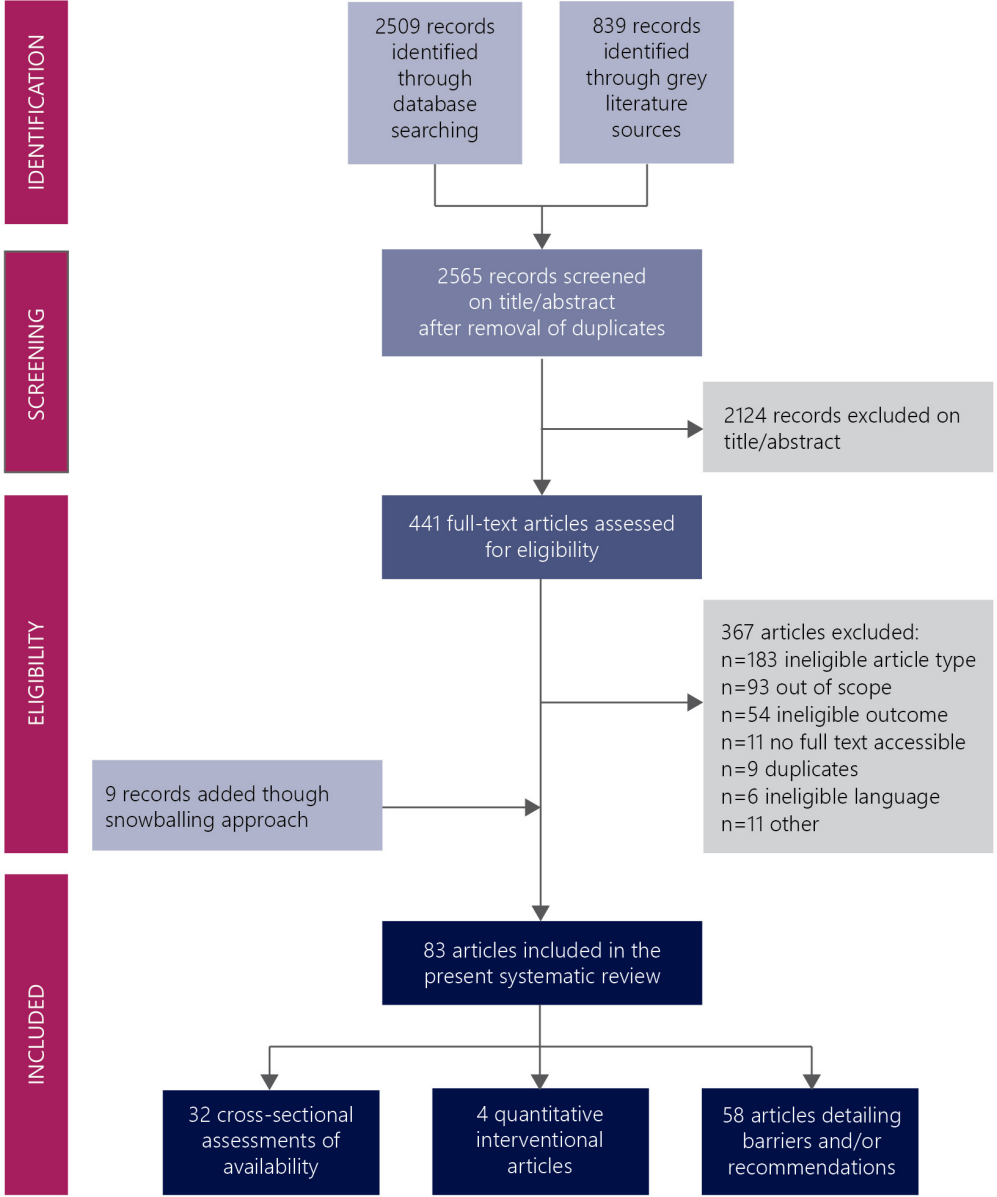
Flow chart of database searches and inclusion process. The databases searched were PubMed, Embase and Web of Science. The search for grey literature sources included the World Bank eLibrary and the WHO Institutional Repository for Information Sharing. Included articles could be placed in multiple categories.

Electronic database searches identified 3348 records. After removal of duplicates and eligibility screening on title/abstract level,^17^ 441 records remained. Following full-text assessment, 74 records met the inclusion criteria for this scoping review, and another 9 were added following the screening of reference lists, resulting in 83 included records (figure 1).

NCDs discussed most often in the included records were cancers (28%), CVDs (16%) and diabetes (18%), frequently within the context of countries in the African Region (40%) (table 1). Notably, a large majority (70%) of eligible literature was published in the last 5 years.

**Table 1:** General characteristics of included studies

	Cross-sectional studies	Interventional studies	Studies detailing barriers/recommendations	Cross-sectional studies also detailing barriers/recommendations	All studies
	n=21	n=4	n=47	n=11	n=83
Type of NCD targeted					
Cancer	4	0	16	3	23 (28%)
Cardiovascular diseases	7	1	4	1	13 (16%)
Diabetes	2	1	10	2	15 (18%)
Mental health conditions	0	0	3	0	3 (4%)
Respiratory diseases	0	0	4	0	4 (5%)
Multiple NCDs	8	2	10	5	25 (30%)
Region*					
African region	11	3	14	5	33 (40%)
Eastern Mediterranean region	5	0	4	1	10 (12%)
European region	0	0	2	1	3 (4%)
Region of the Americas	1	0	8	0	9 (11%)
South-East Asian region	1	1	5	4	11 (13%)
Western Pacific region	0	0	3	0	3 (4%)
Multiple regions/global	3	0	11	0	14 (17%)
Year of publication					
2009–2013	0	1	4	1	6 (7%)
2014–2018	5	0	13	1	19 (23%)
2019–2025†	16	3	30	9	58 (70%)

* Classification based on WHO regions.

† Until May 2025.

NCD, non-communicable disease.

### Cross-sectional assessments of availability

**Figure 2:**
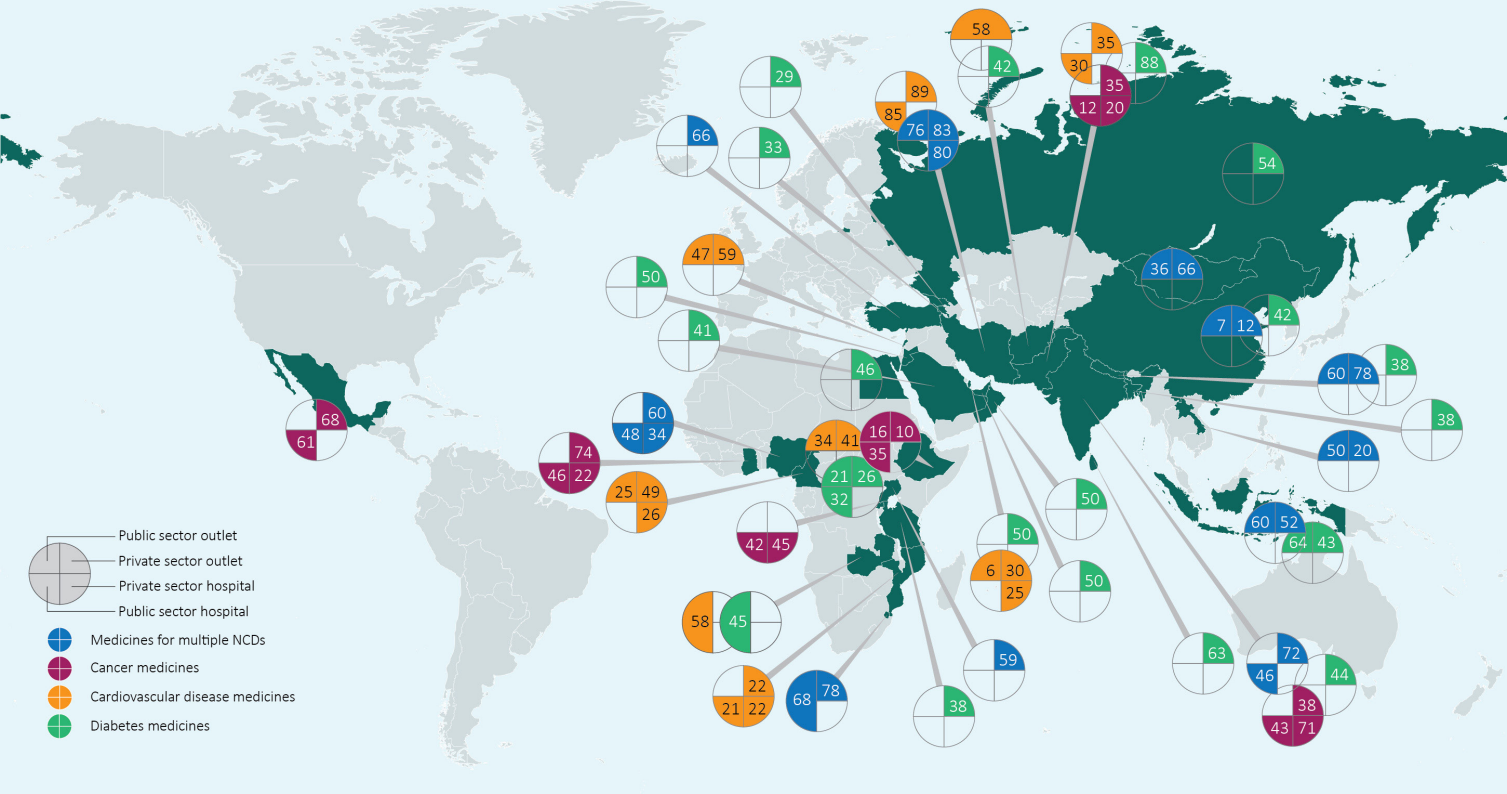
World map of mean availability (%) of NCD medicines. Note: Not all outcomes reported in the individual articles are shown in this figure: results from sectors other than the public, private or mixed sector are not visualised. In the case of multiple outcomes, preference was given to any medicine rather than originator brand (OB) or lowest-priced generics (LPGs) separately. If outcomes for any medicine were not reported, LPG outcomes are represented in this figure. Similarly, when the country of survey and therapeutic area were the same across multiple surveys, preference was given to the most recent surveys. All outcomes are presented in online supplemental annex 2. NCD, non-communicable disease.

Quantitative assessments of availability in the form of cross-sectional surveys in selected health facilities revealed that the mean availability of NCD medicines on the day of the survey was suboptimal (<80%) in 39 of the 46 surveyed countries between 2010 and 2022 (figure 2).^818^,^48^ This deficiency was observed across therapeutic areas, with no apparent improvement over time. Detailed findings and source materials are presented in online supplemental annex 2.

### Quantitative studies on interventions to improve availability of NCD medicines

This scoping review identified four quantitative studies reporting on the effects of interventions intended to increase access to NCD medicines.

Rockers *et al* assessed the impact of the Novartis Access programme on medicine availability and pricing in Kenya.^49^ In elected counties, health facilities could purchase a range of NCD medicines at a wholesale price of US$1 per month. In this cluster-randomised controlled trial, results showed increased availability of amlodipine (adjusted OR (aOR) 2.84, p=0.031) and metformin (aOR 4.78, p=0.011). However, no significant changes in the availability or patient prices of the entire portfolio were observed after 15 months of the programme.

Tran *et al* tested three revolving fund pharmacy models to increase the availability of CVD medicines in Western Kenya.^50^ Fundamentally, these models operate on a revolving fund mechanism following the initial donation or purchasing of medicine stocks, subsequently sustained through revenue from selling the medicines at a small markup sufficient to replenish stocks. Multiple stakeholders were involved, including the Ministry of Health, local leadership, facility leadership and an academic-hospital partnership that established the programme. The availability of eight CVD medicines increased from less than 30% before the intervention to 90% or higher, with all medicines reaching the 80% target for availability across all health system levels. Operational costs of the programme varied from US$6.5 to US$25 per patient per year.

Beran *et al* reported on the Diabetes UK Mozambique Twinning Programme, a partnership between diabetes organisations of both countries.^51^ The intervention included training for health workers, specialists and other stakeholders in the PVC, developing patient education materials, providing policy support, strengthening the diabetes organisation and initiating diabetes research programmes. On repeated measurement of key indicators following 3 years of the intervention, procurement cycles had shortened from 1 year to 9 months, procurement prices and quantities had decreased (the latter due to better planning and reduced wastage), and more insulin was being distributed to peripheral areas. Insulin availability at hospitals increased from 20% to 100%.

The quasi-randomised cluster trial conducted by Pati *et al* assessed the effectiveness of health service optimisation alone or in combination with community platform strengthening in improving access to NCD medicines at the primary care level (PHC) in India.^52^ Health service optimisation (arm A) included training of PHC staff on the diagnosis and management of diabetes and hypertension, the introduction of patient-retained medical records and PHC-based records, and the coordination of different stakeholders to ensure a continuous medicine supply. Community platform strengthening included the dissemination of awareness materials and the forming of patient groups. Following an 18-month intervention period, the mean number of days with available medicines increased by 31.5 and 11·9 days for diabetes and hypertension medicines, respectively, in study arm A compared with the control group. In arm B, the authors found an increase of 17.8 days for antidiabetics and a reduction of 1.5 days for antihypertensives. None of the results were statistically significant.

### Barriers to available NCD medicines and recommendations to improve availability

**Figure 3:**
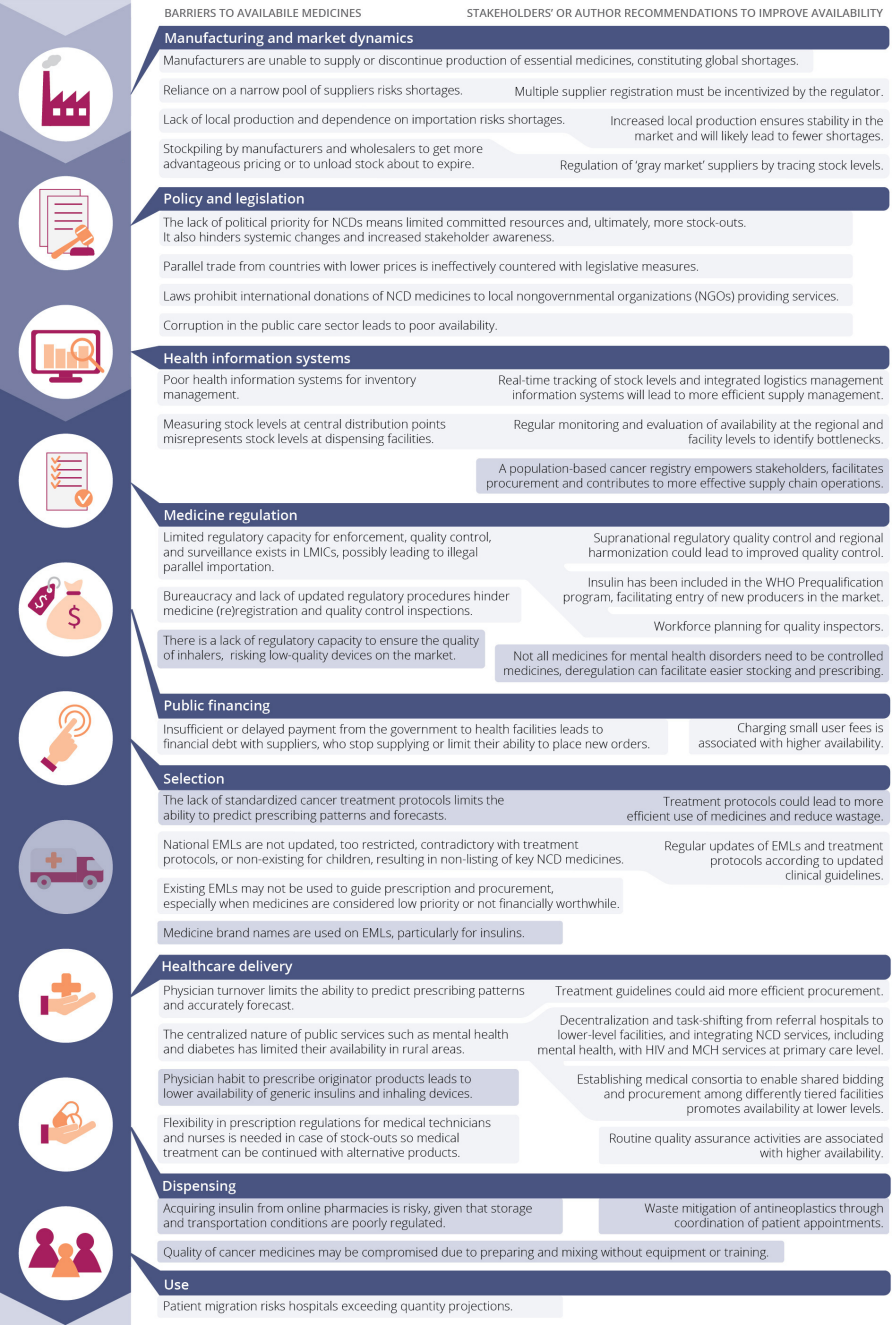
Barriers to NCD medicines’ availability across the supply chain (left) and recommendations to improve availability according to stakeholders and authors (right). Correlated barriers and recommendations are linked. Disease-specific barriers are shown in dark blue. EMLs, Essential Medicines List; LMIC, low-income and middle-income country; MCH, maternal and child health; NCD, non-communicable disease.

A total of 58 records described barriers to availability and/or recommendations to improve availability (figures3  4, online supplemental annex 3).^1922 24^,^26 33 37 39 40 43 48 53^ A description of core barriers and recommendations per PVC component is provided below.

**Figure 4:**
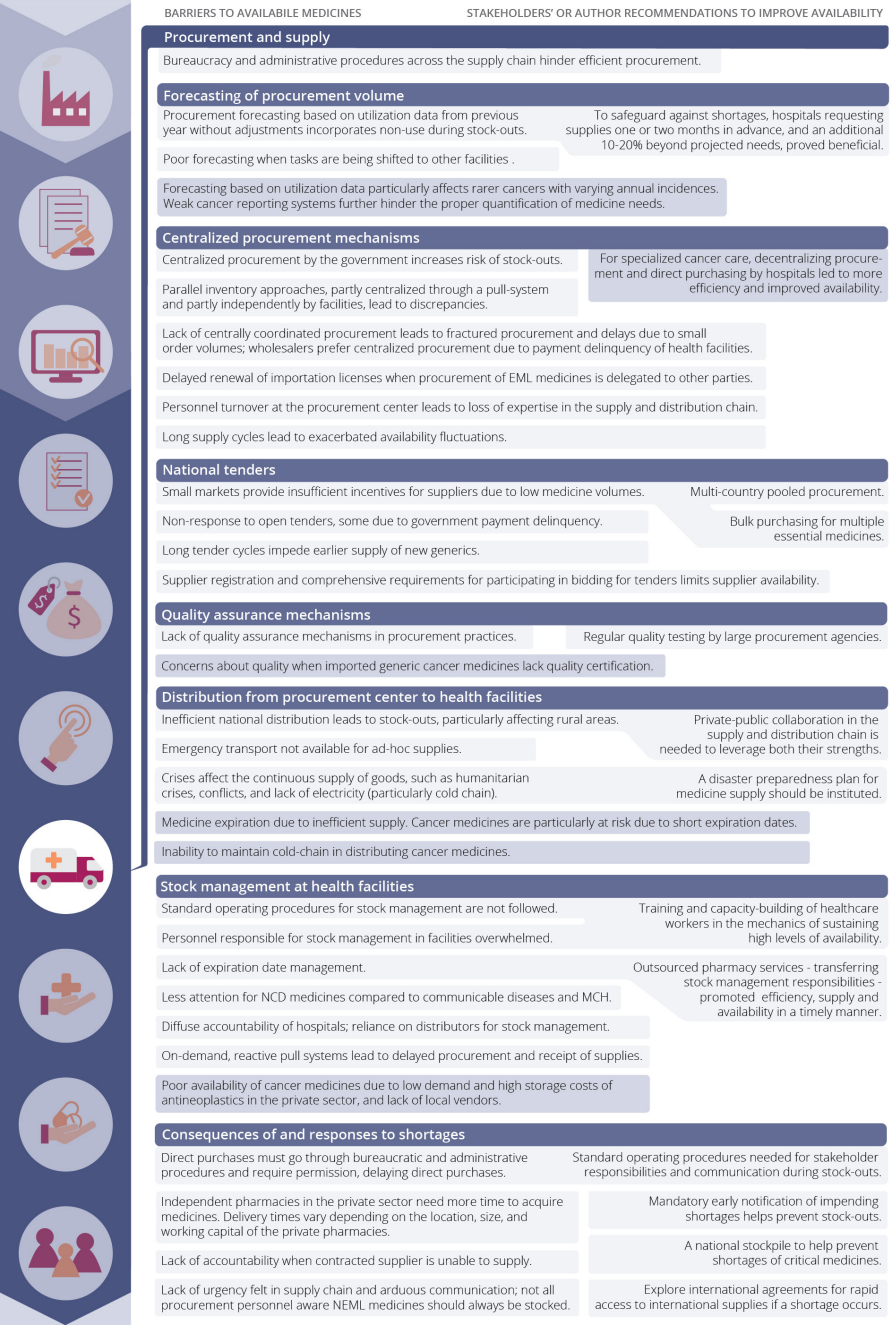
Barriers to available NCD medicines (left) in the component ‘procurement and supply’ and recommendations to improve availability according to stakeholders and authors (right). Correlated barriers and recommendations are linked. Disease-specific barriers are shown in dark blue. MCH, maternal and child health; NCD, non-communicable disease; NEMLs, National Essential Medicines List.

### Manufacturing and market dynamics

Although extending beyond the national context, local interventions may nonetheless be appropriate to limit the influence of global determinants and offset discontinuation of essential NCD generic medicine production.^53^ First, to reduce the risk of shortages due to a country’s reliance on a narrow pool of suppliers,^54^ multiple supplier registration must be incentivised.^55^ Multicountry pooled procurement may be explored to increase suppliers’ interest in supplying to smaller markets,^54 56^ as well as bulk procurement of multiple NCD medicines from the same supplier.^54^ It should also be noted that global market dynamics (ie, shortages) are less likely to affect supply when medicines are produced locally.^5557^,^59^

### Policy and legislation

The lack of political priority given to NCDs means limited resources have been committed to providing access to NCD medicines,^53 57 60^ indirectly contributing to the number of stock-outs. Without increased political priority, the required systemic changes will likely stay out. A lack of good governance and corruption has also been identified as significant barriers to available medicines, particularly in the public health sector.^61^

### Health information systems

Health information systems are crucial in forecasting the required procurement volumes of medicines; lack thereof constitutes a significant barrier, particularly for cancers.^60^ Furthermore, inadequate information systems for tracking stock levels and inventory management contribute to stock-outs.^56^ Real-time tracking across the value chain, and not solely in the central distribution point, is strongly advised.^26 57 62^

### Medicine regulation

Several sources reported a lack of regulatory capacity for enforcement and quality control, impairing a country’s capacity to provide quality medicines.^19 63 64^ This may be particularly relevant for complex pharmaceuticals and devices, such as inhalers; due to the lack of regulatory capacity, low-quality devices may see registration.^65^ Bureaucratic procedures further hamper quality control inspections.^55 64^ The lack of enforcement can also lead to illegal parallel importation, contributing to the problem of falsified and substandard medicines. Supranational regulatory bodies, harmonisation and better workforce planning have been suggested for increased regulatory capacity.^54 64^ Additionally, inclusion of insulin in the WHO Prequalification programme could facilitate entry of new products in local markets.^48^

### Public financing

Without sufficient funds to acquire medicines, procured quantities are unlikely to meet the demand. To increase funds for procurement and improved service delivery, charging small user fees was associated with better availability.^61 66^ Besides insufficient funds, governmental payment delinquency to health facilities can result in the latter going into debt with suppliers, who then stop supplying facilities.^62 67 68^ Updated and expedited administrative procedures are thus essential in reducing the number of stock-outs.

### Selection

National Essential Medicines Lists (NEMLs) and National Standard Treatment Guidelines (NSTGs) are important tools at a country’s disposal to guide the efficient use of limited resources and help predict usage patterns, which is critical in forecasting medicine demand and reducing wastage.^56 68^ The absence of NSTGs, mainly observed for specialised areas such as cancer, should thus be explored.^68^

Multiple sources reported that NEMLs are not updated, too restricted or contradictory with treatment protocols, which results in key NCD medicines not being listed.^6769^,^73^ In addition, some NEMLs list brand names rather than international non-proprietary names, mainly observed for insulins,^43^ contributing to lower availability of generic products. However, even when NEMLs are complete and updated, they may not be used to guide prescription and procurement,^33 74^ especially when specific medicines are considered low priority.^75^

### Procurement and supply

Given its central role in determining NCD medicine availability at the service delivery point, this component was further dissected into smaller elements (figure 4) as outlined below.

#### Forecasting of procurement volume

Correct forecasting of the demand is fundamental to procuring sufficient medicine quantities. However, forecasting based on utilisation data from the previous year could incorporate periods of non-use and underestimate the required quantities when the previous year included periods of stock-outs.^60 68 76 77^ This may particularly affect cancers with varying annual incidences and poor reporting systems.^56 57 78^ Beyond this, hospitals reported positive experiences when acquiring 10%–20% more than the forecasted need and ordering new stocks 1–2 months before stocks were estimated to run out.^67^

#### Centralised procurement mechanisms

Stakeholders and authors reported varying experiences with centralised procurement mechanisms: ineffective central procurement processes were associated with more stock-outs,^67^ but decentralised and fractured procurement were also linked to unavailability and procurement delays,^57 79^ and a combination of both through parallel inventory approaches was not seen as effective either.^60^ Nonetheless, there is a theoretical argument for centralised procurement, which can use the limited resources more efficiently and reduce duplication of efforts. An effective central procurement centre with experienced procurement and inventory management personnel is essential therein.^56^ Additionally, shorter supply cycles from a central procurement centre to health facilities may mitigate stock-out risks.^40^ Despite these arguments, decentralised procurement could result in more accurate forecasting and quicker supply through direct purchasing for highly specialised care areas in a limited number of facilities across the country, such as for cancers.^60^

#### National tenders

National tenders are an important instrument in efficient procurement of medicines. Still, stringent supplier registration and comprehensive requirements for participation in these tenders were associated with lower supplier availability and, by extension, lower medicine availability.^53 56^ Suppliers may also be unwilling to participate in tenders due to governmental payment delinquency in previous rounds.^57 80^

#### Quality assurance

Besides the national regulator, procurers—particularly large procurement agencies—play a key role in assuring the quality of medicines on the market. Regular quality testing of procured stocks at this level is critical,^56 57 81^ and generic medicines with quality certifications should be prioritised.^75^

#### Distribution from procurement centre to health facilities

Inefficient national distribution was identified as an important barrier affecting rural areas^58 76 82^ as well as products with short expiration dates^54^ and the inability to maintain cold chain.^53^ In this regard, public–private partnerships in distributing to rural areas—leveraging both sectors’ strengths—were suggested to achieve better distribution.^83^ In addition, reactive pull systems lead to delayed supply.^54 56^ Efficient distribution may be at further risk when human or environmental crises occur.^71 79^ Therefore, a disaster management plan for medicine supply is advised.^84^,^86^

#### Stock management at health facilities

Those responsible for stock management at individual health facilities are often overwhelmed with other tasks, fail to follow standard operating procedures, do not manage expiration dates effectively, rely on others to manage stocks, or fail to prioritise NCD medicines as they underestimate the importance of uninterrupted NCD care.^58 60 62 87^ Stock managers’ further training and capacity building are essential in reducing stock-outs.^83^ Alternatively, outsourced pharmacy management, transferring responsibilities to an external party, was associated with improved efficiency.^67^

#### Consequences of and responses to shortages

Direct purchasing may be used to acquire the required medicines when stock-outs occur. This pathway is associated with considerable delays, as the required permission is associated with lengthy and bureaucratic administrative procedures.^56 67^ The perceived lack of urgency felt by and arduous communication with procurement professionals must be addressed.^53^ Standard operating procedures for stock-out situations could considerably reduce delays.^56^

### Healthcare delivery

At the service delivery level, prescribers play a critical role in the availability of the products they prescribe; products prescribed more frequently are generally more likely to be stocked. Their inclination to prescribe brand-name insulins and oral anti-asthmatics results in lower availability of generic insulins and inhaling devices.^39 58 71 88^ Beyond this, quick prescriber turnover at health facilities limits the procurer’s ability to forecast which medicines to acquire.^68^

On an organisational level, service delivery for many NCDs is still centralised, resulting in lower availability of NCD medicines at rural health facilities.^61 89^ Decentralisation of NCD management, such as diabetes, CVDs and mental health disorders, is essential to improve medicine availability at health facilities.^90 91^ Medical consortia consisting of differently tiered health facilities could facilitate shared bidding and promote availability at lower levels.^92^ Finally, routine quality assurance activities at health facilities were associated with a higher availability of medicines.^61 66^

### Dispensing

Stakeholders reported opportunities to mitigate waste of antineoplastics by coordinating patient appointments: by planning appointments on the same day—and thus preparing chemotherapy for multiple patients simultaneously—waste and stock-outs can be reduced.^57 93^ Additionally, increased attention should be paid to the safe preparation of chemotherapies, to prevent compromised quality due to preparation and mixing without adequate equipment or training.^53^

### Use

Whether the quantities on the shelf meet the demand depends on the supply-demand equilibrium. Patient migration may destabilise this equilibrium: when patients migrate towards other health facilities or regions for treatment when medicines are unavailable in their facility, the demand increases at the receiving facility, exceeding their projected demand and procured quantities.^67^

## Discussion

By mapping data from over 80 qualitative and quantitative studies, we provide evidence of the global unavailability of NCD medicines and a comprehensive overview of the current processes and procedures across the PVC that determine (un)availability. Although most identified barriers pertain to supply chain inefficiencies, these findings also emphasise the interlinkage of the different processes, stakeholders and various health system building blocks that directly or indirectly affect the availability of essential NCD medicines.

Some identified deficiencies and possible interventions do not require significant legislative changes and can be resolved through updated procedures and guidelines. To illustrate, updating NEMLs to include critical NCD medicines or amending forecasting procedures to exclude periods of stock-outs can be effective tools to improve availability. Suitable methodologies and tools for predicting volumes and costs of cancer medicines have been proposed^100 101^ and may be adapted beyond cancer to strengthen access to all essential NCD medicines. Beyond these initial, lower-complexity interventions, considerable long-term financial commitment will be needed for more fundamental progress, in which modernised and integrated stock management systems and increased availability and training of the health workforce are vital. The latter is also key in decentralising health services to lower-level health facilities, identified in this review as a critical approach to improving the availability of NCD medicines. This was confirmed in the Rwandan context, where management of severe NCDs was successfully decentralised, and medicine availability increased.^102^ Additionally, the supply chain may require structural rethinking. For example, in a randomised experiment in Zambia in which the number of tiers in the medicine distribution system was reduced, enabling more direct distribution from a central procurement centre to individual health facilities, the duration and frequency of stock-outs decreased significantly.^103^ Finally, opportunities for international collaboration and harmonisation should be explored for optimal use of limited financial and human resources. Such efforts could include regional regulatory harmonisation,^78^ further expansion of the WHO Prequalification programme to include more NCD medicines,^104^ or regional pooled procurement.^78^ Recent evidence confirms that the latter can significantly lower drug prices and reduce delivery delays in LMICs, thereby improving not only affordability but also availability of NCDs.^105^ However, it may increase procurement lead times, underlining trade-offs that must be considered in NCD procurement planning.

According to Beran *et al*, a positive political change contributed significantly to the success of the Diabetes UK Mozambique Twinning Programme, confirming the importance of political commitment to NCD management.^51^ In addition to affirming some of the obstacles and recommendations identified from other included records (ie, frequent staff turnover, lack of priority given to NCD management at PHC level^51^), the four quantitative, interventional studies provide insight into the ingredients for a successful intervention. First, effective interventions engaged multiple stakeholder groups across the supply chain, including but not limited to the national and local government, supply chain workers, health facility workers, civil society organisations and patients.^51^ In contrast, an intervention without broader strategies was not translated into more accessible essential medicines for patients.^49^ Effective interventions also addressed the need for increased financing, required to sustain stock levels by charging small patient fees.^50 51^ Without providing additional financial resources, health service optimisation failed to improve NCD medicine availability.^52^

Our study confirms several of the barriers and opportunities identified in previous reports mapping challenges in procurement and supply chains, though not specific to NCD medicines.^106 107^ However, these resources also highlight additional recommendations of potential benefit to NCD medicines, such as the need for improved funding for the supply chain operating costs, outsourced medicine transport services by contracting third parties to distribute stocks, and creating market competition for the central procurement centre through decentralisation to smaller regions, districts or even individual facilities.^107^ Vice versa, the barriers and recommendations identified in our review likely have broader implications for other essential medicines, and synergies should be explored.

This study is subject to several limitations. First, we excluded studies measuring stock-outs rather than mean or median availability to ensure comparability of the data. Although this could have increased the number of eligible studies, it is unlikely that this would reveal a different pattern of availability. Second, our search included major therapeutic areas (including CVDs, cancer, diabetes, etc), but we did not exhaustively search for all individual disorders. Similarly, we identified little empirical literature on supply chain interventions. Given that studies describing such interventions may be published in non-medical scientific journals, the databases used in this scoping review may not adequately cover these studies. Third, compartmentalising the PVC and differentiation between various access dimensions is inherently complex and subject to interpretation. However, the level of detail required to understand and address the challenges to available NCD medicines is missing without this compartmentalisation. Fourth, the scope of the present review was limited to national barriers and recommendations, and the availability of existing medicines. Given that interventions for improved availability of NCD medicines often targeted multiple dimensions of access (ie, affordability), there was some subjectivity in selecting only those studies primarily focused on availability. Our approach also excluded factors such as market access, often considered part of the availability dimension in high-income countries.^108^

In conducting this scoping review, several implications for future research were identified. Primarily, although we were able to create a broad overview of availability challenges, the included studies rarely provided in-depth information about supply chain issues; availability issues were often overlooked compared with the pricing and affordability of medicines. Although the general recommendation to reduce supply chain inefficiencies was often given, the details to help understand these inefficiencies and how they can be addressed were often missing. In-depth qualitative studies mapping barriers to medicine availability were exclusive to the cancer domain but would also be of significant benefit to other therapeutic areas. Similarly, of the studies reporting on cross-sectional availability assessments, only a few also unearthed specific reasons for limited availability when scores were suboptimal. We, therefore, urge researchers to include this in the scope of their future studies. Lastly, we identified very little literature describing barriers to medicines for the management of mental health disorders and barriers to paediatric NCD medicines other than in the domain of childhood cancer. This evidence void must be addressed to ensure all NCDs and populations benefit from health systems strengthening.

## Conclusions

This scoping review provides insight into the processes and procedures that currently determine the availability of NCD medicines in LMIC, demonstrating that barriers occur across the PVC and are not limited to the supply and distribution chain only. Although this overview increases our understanding of where problems can arise, which stakeholders are involved and potential solutions, our findings provide a starting point for improving availability only. There is an evident need for in-depth information on barriers to available NCD medicines, in which root causes of unavailability are carefully explored. Nonetheless, this evidence highlights the need for political commitment to NCDs, broad stakeholder involvement, integrated stock management systems and increased human resources to make NCD medicines more accessible to patients. Appropriate policy interventions should be determined on a case-by-case basis, with the recommendations in this review serving as a potential guide.

## Supplementary material

10.1136/bmjgh-2025-019634 online supplemental file 1

## Data Availability

All data relevant to the study are included in the article or uploaded as supplementary information.
