# Supplementary material for: Availability of essential medicines for non-communicable diseases: a scoping review of challenges and opportunities
Source: BMJ Glob Health. 2025 Nov 27;10(11):e019634. doi: 10.1136/bmjgh-2025-019634 (PMC12666150; doi:10.1136/bmjgh-2025-019634)
Supplement: online supplemental file 1 [file bmjgh-10-11-s001.pdf]

Annex 1

Search string

Medicines
Medicines OR medicine OR medication OR medications OR medicament OR pharmaceuticals OR pharmaceutical OR biopharmaceutical OR biopharmaceuticals OR drug OR drugs OR generic OR generics OR biosimilar OR biosimilars OR insulin

AND

Non-communicable diseases
Non-communicable disease OR non-communicable diseases OR non-communicable disease OR non-communicable diseases OR NCD OR NCDs OR chronic disease OR chronic diseases OR non-infectious disease OR non-infectious disease OR non-infectious diseases OR non-infectious diseases OR cardiovascular diseases OR cardiovascular diseases OR cancer OR oncology OR diabetes OR COPD OR chronic obstructive pulmonary disease OR asthma OR mental health OR epilepsy

AND

Availability
available OR availability OR present OR access OR volume OR quantity OR shortage OR out of stock OR out-of-stock OR procurement OR supply OR supply chain OR shelf OR distribution

This query was modified for the World Bank eLibrary and the WHO IRIS databases, which have more limited search functionalities.

We searched for these core elements in the record title to keep the number of hits manageable.

Annex 2

Details on cross-sectional assessments of availability

Table S1 Details on cross-sectional assessments of availability.

Country	Therapeutic area	Year of survey	Sector	Facility type‡	Mean availability (%)		
					Mixed/ unspecified	OB	LPG
Afghanistan	Diabetes [1]	2018	Private	Pharmacy	42	50†	33†
	CVD [2]	2020	Mixed	Pharmacy	46		
Armenia	Diabetes [1]	2018	Private	Pharmacy	33	25†	40†
Bangladesh	Diabetes [1]	2018	Private	Pharmacy	38	0†	75†
Cameroon	CVD [3]	2016	Private	Hospital	26		
			Public	Pharmacy	25		
			Private	Pharmacy	49		
			Mission	Pharmacy	44†		
China	Multiple NCD [4]	2012	Public	Pharmacy		3†+	7+
			Private	Pharmacy		10†+	12+
	Diabetes [1]	2018	Private	Pharmacy	42	42†	42†
Egypt	Diabetes [1]	2018	Private	Pharmacy	46	17†	75†
Ethiopia	Cancer [5]	2022	Public	Hospital	35		
			Private	Pharmacy	10		
			Public	Pharmacy	16		
	CVD [6]	2022	Private	Pharmacy	33†		
			Public	Hospital	28†		
	Diabetes [7]	2022	Public	Hospital			32
			Public	Pharmacy			21
			Private	Pharmacy		5	26
	Thyroid dysfunction [7]	2022	Public	Hospital			25†
			Public	Pharmacy			33†
			Private	Pharmacy		1†	21†
	Diabetes [8]	2022	Public	Hospital			6†+
			Public	Pharmacy			0†+
			Private	Pharmacy		0†+	47†+
	CVD [9]	2022	Public	Pharmacy			34
			Private	Pharmacy			41
Georgia	Diabetes [1]	2018	Private	Pharmacy	29	33†	25†
Ghana	Cancer [10]	2020	Public	Hospital	27†		
			Private	Pharmacy	75†		
	Cancer [11]	2021	Public	Hospital	46		
			Private	Hospital	22		
			Private	Pharmacy	74		
India	Multiple NCD [4]	2011	Public	Pharmacy		0†+	41†+
			Private	Pharmacy		38†+	90†+
	Cancer [12]	2018	Private	Pharmacy	38		
			Private	Hospital	71		

			Public	Hospital	43		
	Diabetes [1]	2018	Private	Pharmacy	44	25†	63†
	Multiple NCD [13]	2020	Public	Hospital	46		
			Other	Pharmacy	65†		
			Private	Pharmacy	72		
Indonesia	Multiple NCD [4]	2010	Public	Pharmacy		2†+	60+
			Private	Pharmacy		16†+	52+
	Diabetes [14]	2022	Public	Pharmacy	64		
			Private	Pharmacy	43		
Iran	Multiple NCD [15]	2014	Public	Pharmacy	76		
			Private	Pharmacy	83		
			Private	Hospital (outpatient)	80		
	CVD [16]	2015	Public	Hospital	85		
			Public	Hospital (outpatient)	88		
			Private	Pharmacy	89		
Jordan	Diabetes [1]	2018	Private	Pharmacy	50	50†	50†
	Multiple NCD [4]	2013	Public	Pharmacy		20†+	50+
			Private	Pharmacy		38†+	20+
Lebanon	CVD [17]	2016	Public	Pharmacy		0†	47
			Private	Pharmacy		70†	59
Mexico	Cancer [18]	2017	Public	Hospital	61		
			Private	Pharmacy	68		
Mongolia	Multiple NCD [4]	2012	Public	Pharmacy		23+	36†+
			Private	Pharmacy		37+	66†+
Mozambique	CVD [19]	2018	Private	Pharmacy	22		
			Private	Hospital	22		
			Public	Hospital	21		
Nepal	Multiple NCD [20]	2015	Public	Pharmacy	60		
			Private	Pharmacy	78		
	Diabetes [1]	2018	Private	Pharmacy	38	0†	75†
Nigeria	Multiple NCD [21]	2019	Private	Pharmacy	60		
			Public	Hospital	48		
			Private	Hospital	34		
Oman	Diabetes [1]	2018	Private	Pharmacy	50	33†	67†
Pakistan	Cancer [22]	2016	Public	Hospital		31†	12
			Private	Hospital		72†	20
			Private	Pharmacy		59†	35
	Diabetes [1]	2018	Private	Pharmacy	88	75†	100†
	CVD [23]	2019	Public	Hospital	30		
			Private	Pharmacy	35		
Qatar	CVD [17]	2016	Public	Pharmacy		82†	6
			Private	Pharmacy		65†	30
			Private	Hospital (outpatient)		55†	25
	Diabetes [1]	2018	Private	Pharmacy	50	58†	42†

Russian Federation	Diabetes [1]	2018	Private	Pharmacy	54	58†	50†
Rwanda	Cancer [24]	2022	Public	Hospital	42		
			Private	Hospital	45		
Saudi Arabia	Diabetes [1]	2018	Private	Pharmacy	41	63†	19†
Sri Lanka	Multiple NCD [25]	2013	Public	Hospital (outpatient)		50-80†	
			Private	Hospital (outpatient)		50-80†	
			Public	Pharmacy		80-100†	
			Private	Pharmacy		50-80†	
	Diabetes [1]	2018	Private	Pharmacy	63	67†	58†
Swaziland	Multiple NCD [26]	2013	Private	Pharmacy		80†	78
			Public	Mixed		40†	68
Tanzania	Diabetes [1]	2018	Private	Pharmacy	38	25†	50†
Turkey	Multiple NCD [27]	2021	Private	Pharmacy		87†	66
Uganda	Multiple NCD [28]	2016	Mixed	Mixed	44†		
	Multiple NCD [29]	2019	Private	Pharmacy	59		
United Arab Emirates	Diabetes [1]	2018	Private	Pharmacy	50	50†	50†
Zambia	CVD [30]	2016	Public	Mixed	58		
	Diabetes [1]	2016	Public	Mixed	45		
	Multiple NCD [26]	2019	Private	Pharmacy		30-50†	
Global	Multiple NCD [32]	2001-2006	Public	Pharmacy	42†		
			Private	Pharmacy	69†		

CVD = cardiovascular diseases; NCD = non-communicable diseases; OB = originator brand; LPG = lowest-priced generic.

†Not shown in Figure 2. When applicable, preference was given to mixed outcomes over OB or LPGs. If not reported, LPGs outcomes were represented in Figure 2. Similarly, when the country of survey and therapeutic area were the same, preference was given to the most recent outcomes.

‡Pharmacy or other medicines outlet at the first level of care.

$^{+}$Median rather than mean availability.

References

1. Babar ZUD, Ramzan S, El-Dahiyat F, Tachmazidis I, Adebisi A, Hasan SS. The Availability, Pricing, and Affordability of Essential Diabetes Medicines in 17 Low-, Middle-, and High-Income Countries. Front Pharmacol. 2019 Nov 19;10:1375.
2. Kokabisaghi F, Hashemi-Meshkini A, Obewal A, Ghavami V, Javan-Noughabi J, Shabanikiya H, et al. Availability and affordability of cardiovascular medicines in a major city of Afghanistan in 2020. DARU J Pharm Sci. 2022 Dec;30(2):343-50.
3. Dzudie A, Njume E, Abanda M, Aminde L, Hamadou B, Dzekem B, et al. Availability, cost and affordability of essential cardiovascular disease medicines in the south west region of Cameroon: Preliminary findings from the Cameroon science for disease study. PLoS One. 2020 Mar;15(3):e0229307.
4. Wang HP, Sun Q, Vitry A, Nguyen TA. Availability, Price, and Affordability of Selected Essential Medicines for Chronic Diseases in 11 Countries of the Asia Pacific Region: A Secondary Analysis. Asia Pac J Public Health. 2017 May;29(4):268-277.
5. Alemu BA, Hailemariam FH. Price, Availability and Affordability of Anti-Cancer Medicines in Addis Ababa, Ethiopia. Risk Manag Healthc Policy. 2022 Dec 29;15:2421-2433.
6. Limenh LW, Tessema TA, Sendekie AK, Simegn W, Ayenew W, Mitku ML, et al. Availability and Price Variations of Commonly Used Cardiovascular Medicines at Community and Hospital Pharmacies in Gondar Town, Northwest Ethiopia. Biomed Res Int. 2024 Sep 17;2024:6551639.
7. Mohammed SA, Mengesha HY, Andualem A, Seid E, Assefa GM. Availability, price, and affordability of diabetes mellitus and thyroid dysfunction medicines in South Wollo zone, Northeast Ethiopia. BMC Health Serv Res. 2024 Nov 19;24(1):1434.
8. Deressa HD, Abuye H, Adinew A, Ali MK, Kebede T, Habte BM. Access to essential medicines for diabetes care: availability, price, and affordability in central Ethiopia. Glob Health Res Policy. 2024 Apr 7;9(1):12.
9. Masresha R, Habte F, Senbeta MF, Baye AM, Kassaw C, Beyene MG. Availability, price and affordability of essential medicines for managing cardiovascular disease in Addis Ababa, Ethiopia. Sci Rep. 2024 Dec 28;14(1):31357.
10. Mensah KB, Mensah ABB, Bangalee V, Padayachee N, Oosthuizen F. Evaluating essential medicines for treating childhood cancers: availability, price and affordability study in Ghana. BMC Cancer. 2021 Jun;21(1):683.
11. Mattila PO, Biritwum RB, Babar ZUD. A comprehensive survey of cancer medicines prices, availability and affordability in Ghana. PLOS ONE. 2023 May;18(5).
12. Faruqui N, Martiniuk A, Sharma A, Sharma C, Rathore B, Arora RS, et al. Evaluating access to essential medicines for treating childhood cancers: a medicines availability, price and affordability study in New Delhi, India. BMJ Glob Health. 2019 Apr 23;4(2):e001379.
13. Satheesh G, Sharma A, Puthean S, Ansil TPM, Jereena E, Raj Mishra S, et al. Availability, price and affordability of essential medicines for managing cardiovascular diseases and diabetes: a statewide survey in Kerala, India. Trop Med Int Health. 2020 Dec;25(12):1467-79.
14. Ramadaniati HU, Anggriani Y, Lepeska M, Beran D, Ewen M. Availability, price and affordability of insulin, delivery devices and self-monitoring blood glucose devices in Indonesia. PLoS One. 2024 Oct 3;19(10):e0309350.
15. Heidari E, Varmaghani M, Abdollahiasl A. Availability, pricing and affordability of selected medicines for noncommunicable diseases. East Mediterr Health J. 2019 Oct;25(7):473-480.
16. Farahani AV, Salamzadeh J, Rasekh HR, Najafi S, Mosadegh V. The Availability and Affordability of Cardiovascular Medicines for Secondary Prevention in Tehran Province

(Iran). Iran J Pharm Res. 2018 Winter;17(Suppl):64-72.

17. Rida NA, Ibrahim MIM, Babar ZUD. Relationship between pharmaceutical pricing strategies with price, availability, and affordability of cardiovascular disease medicines: surveys in Qatar and Lebanon. BMC Health Serv Res. 2019 Dec;19(1):973.

18. Moye-Holz D, Ewen M, Dreser A, Bautista-Arredondo S, Soria-Saucedo R, van Dijk JP, et al. Availability, prices, and affordability of selected essential cancer medicines in a middle-income country - the case of Mexico. BMC Health Serv Res. 2020 May;20(1):424.

19. Jessen N, Sharma A, Jones J, Auala T, Boladuadua S, Jingi A, et al. Access to Essential Medicines and Diagnostic Tests for Cardiovascular Diseases in Maputo City, Mozambique. Glob Heart. 2023 Feb;18(1):8.

20. Khanal S, Veerman L, Ewen M, Nissen L, Hollingworth S. Availability, Price, and Affordability of Essential Medicines to Manage Noncommunicable Diseases: A National Survey From Nepal. Inquiry. 2019 Jan-Dec;5:46958019887572.

21. Osuafor NG, Ukwe CV, Okonta M. Evaluation of availability, price, and affordability of cardiovascular, diabetes, and global medicines in Abuja, Nigeria. PLoS One. 2021 Aug;16(8):e0255567.

22. Sarwar MR, Iftikhar S, Saqib A. Availability of anticancer medicines in public and private sectors, and their affordability by low, middle and high-income class patients in Pakistan. BMC Cancer. 2018 Jan;18(1):14.

23. Saeed A, Saeed F, Saeed H, Saleem Z, Yang CJ, Chang J, et al. Access to Essential Cardiovascular Medicines in Pakistan: A National Survey on the Availability, Price, and Affordability, Using WHO/HAI Methodology. Front Pharmacol. 2021 Jan;11:595008.

24. Rurangwa C, Ndayisenga J, Sezirahiga J, Nyirimigabo E. Availability and affordability of anticancer medicines at cancer treating hospitals in Rwanda. BMC Health Serv Res. 2023 Jun;23(1):717.

25. Dabare PR, Wanigatunge CA, Beneragama BH. A national survey on availability, price and affordability of selected essential medicines for non communicable diseases in Sri Lanka. BMC Public Health. 2014 Aug 8;14:817.

26. Mhlanga BS, Suleman F. Price, availability and affordability of medicines. Afr J Prim Health Care Fam Med. 2014 Jun 24;6(1):E1-6.

27. Özler G, Işik O. Prices, availability and affordability of selected essential medicines for chronic diseases in Türkiye. East Mediterr Health J. 2023 Nov;26(11):850-60.

28. Armstrong-Hough M, Sharma S, Kishore SP, Akiteng AR, Schwartz JI. Variation in the availability and cost of essential medicines for non-communicable diseases in Uganda: A descriptive time series analysis. PLoS One. 2020 Dec;15(12):e0241555.

29. Isadru VR, Nanyonga RC, Alege JB. Health Facilities' Readiness to Manage Hypertension and Diabetes Cases at Primary Health Facilities in Bidibidi Refugee Settlement, Yumbe District, Uganda. J Trop Med. 2021 Jan 22;2021:1415794.

30. Kalungia CA, Mwale M, Sondashi IS, Mweetwa B, Yassa P, Kadimba G. Availability of Essential Antihypertensive and Antidiabetic Medicines in Public Health Facilities in Lusaka District, Zambia. Med J Zambia. 2017;44(3):140-148.

31. Kaiser AH, Hehman L, Forsberg BC, Simangolwa WM, Sundewall J. Availability, prices and affordability of essential medicines for treatment of diabetes and hypertension in private pharmacies in Zambia. PLoS One. 2019 Dec;14(12):e0226169.

32. Cameron A, Roubos I, Ewen M, Mantel-Teeuwisse AK, Leufkens HG, Laing RO. Differences in the availability of medicines for chronic and acute conditions in the public and private sectors of developing countries. Bull World Health Organ. 2011 Jun 1;89(6):412-21.

Annex 3

Source materials for identified barriers and recommendations

Table S2 Source materials for identified barriers and recommendations.

Barrier	Recommendations
**Manufacturing and market dynamics**	
Manufacturers are unable to supply or discontinue production of essential medicines, constituting global shortages [1, 2].	
Reliance on a narrow pool of suppliers risks shortages [3].	Multiple supplier registration must be incentivized by the regulator [4].
Lack of local production and dependence on importation risks shortages [5-7].	Increased local production ensures stability in the market [8] and will likely lead to fewer shortages [9].
Stockpiling by manufacturers and wholesalers to get more advantageous pricing or to unload stock about to expire [6, 7]	Regulation of 'gray market' suppliers by tracing stock levels [6, 10].
**Policy and legislation**	
The lack of political priority for NCDs means limited committed resources and, ultimately, more stock-outs. It also hinders systemic changes and increased stakeholder awareness [2, 9, 11].	
Parallel trade from lower-priced countries is ineffectively countered with legislative measures [12].	
Laws prohibit international donations of NCD medicines to local nongovernmental organizations (NGOs) that provide care services [5].	
Corruption in the public care sector leads to poor availability [13].	
**Health information systems**	
Poor health information systems for inventory management [14].	Real-time tracking of stock levels and integrated logistics management information systems will lead to more efficient supply management [9, 14-17].
Measuring stock levels at central distribution points misrepresents stock levels at dispensing facilities [17].	Regularly monitor and evaluate availability at the regional and facility levels to identify bottlenecks [18].
	A population-based cancer registry empowers stakeholders, facilitates procurement and contributes to more effective supply chain operations [11].
**Medicine regulation**	
Limited regulatory capacity for enforcement, quality control, and surveillance exists in LMICs, possibly leading to illegal parallel importation [19-21].	Supranational regulatory quality control and regional harmonization could lead to improved quality control [3, 22].
	Insulin has been included in WHO prequalification program, facilitating entry or new producers in the market [23].
	Workforce planning for quality inspectors [21].
Bureaucracy and lack of updated regulatory procedures hinder medicine (re)registration [4] and quality control inspections [21].	
There is a lack of regulatory capacity to ensure the quality of inhalers, which risks low-quality devices on the market [24].	
	Not all medicines for mental health disorders need to be controlled medicines. Deregulation can facilitate easier stocking and prescribing [25].

**Public financing**	
Insufficient or delayed reimbursement/payment from the government to health facilities leads to financial debt with suppliers, who stop supplying or limit their ability to place new orders [1, 15, 26].	
	Charging small user fees is associated with higher availability [13, 27].
**Selection**	
National essential medicines lists are not updated, too restricted, contradictory with treatment protocols, or non-existing for children, resulting in the non-listing of key NCD medicines [1, 28-33].	Regular updates of essential medicines lists and treatment protocols according to updated clinical guidelines [1].
Existing EMLs may not be used to guide prescriptions and procurement [34], especially when medicines are considered low priority or not financially worthwhile [35].	
The lack of standardized cancer treatment protocols limits the ability to predict prescribing patterns and forecasts [26].	Treatment protocols could lead to more efficient use of medicines and reduce wastage [14].
Medicine brand names are used on EMLs, particularly for insulins [36, 37].	
**Procurement and supply**	
Bureaucracy and administrative procedures across the supply chain hinder efficient procurement [4, 7, 38].	
Procurement volume forecasting	
Procurement forecasting based on utilization data from the previous year without adjustments incorporates non-use during stock-outs [11, 26, 42, 43].	To safeguard against shortages, hospitals requesting supplies one or two months in advance, and an additional 10-20% beyond projected needs, proved beneficial [1]
Poor forecasting when tasks are being shifted to other facilities [15].	
Forecasting based on utilization data particularly affects rarer cancers with varying annual incidences [14]. Weak cancer reporting systems further hinder the proper quantification of medicine needs [9, 22].	
Centralized procurement mechanisms	
Centralized procurement by the government increases the risk of stock-outs [1].	For specialized care, decentralizing procurement and direct purchasing by hospitals led to more efficiency and improved availability [11].
Parallel inventory approaches, partly centralized through a pull-system and partly independently by facilities, lead to discrepancies [11].	
A lack of centralized and nationally coordinated procurement and fractured procurement leads to delays due to small order volumes, and wholesalers prefer centralized procurement due to payment delinquency of individual facilities [9, 44].	
Personnel turnover at the procurement center leads to losing expertise in the supply and distribution chain [14].	
Long supply cycles lead to exacerbated availability fluctuations [16].	
Delayed renewal of importation licenses when procurement of EML medicines is delegated to other parties [35].	
National tenders	
Supplier registration and comprehensive requirements for participating in tender bidding limits supplier availability [2, 14].	
Non-response to open tenders, some due to government payment delinquency [9, 45].	

Long tender cycles impede earlier supply of new generics [Joosse].	
Small markets provide insufficient incentives for suppliers due to low medicine volumes [3].	Multi-country pooled procurement [2, 3, 14, 22].
	Bulk purchasing for multiple essential medicines [9].
Quality assurance mechanisms	
Lack of quality assurance mechanisms in procurement practices [9, 14].	Regular quality testing by large procurement agencies [46].
Concerns about quality when imported generic cancer medicines lack quality certification [35].	
Distribution from central procurement center to health facilities	
Inefficient national distribution leads to stock-outs, particularly affecting rural areas [7, 42, 47, 48].	Private-public collaboration in the supply and distribution chain is needed to leverage both strengths [18].
Inability to maintain cold chain in distributing cancer medicines [2].	
Emergency transport is not available for ad-hoc supplies [15].	
Crises affect the continuous supply of goods, such as humanitarian crises, conflicts, and lack of electricity (particularly cold chain) [7, 29].	A disaster preparedness plan for medicine supply should be instituted [49, 50].
Medicine expiration due to inefficient supply [3]. Cancer medicines at particular risk due to short expiration dates [7].	
Stock management at health facilities	
Standard operation procedures for stock management are not followed [15].	Training and capacity building of healthcare workers in the mechanics of sustaining high levels of availability [18].
Personnel responsible for stock management in facilities are overwhelmed [15].	
Less attention for NCD medicine stocks compared to communicable diseases and MCH [51].	
Lack of expiration data management [11].	Outsourced pharmacy services - transferring stock management - promoted efficiency, supply and availability in a timely manner [1].
Diffuse accountability of hospitals due to reliance on distributors for stock management [7]	
On-demand, reactive pull systems lead to delayed procurement and receipt of supplies [3, 14].	
Poor availability due to low demand and high storage costs of anti-neoplastics in the private sector, and lack of local vendors [7, 39-41].	
Consequences of and responses to shortages	
Direct purchases must go through bureaucratic and administrative procedures and require permission, delaying direct purchases [1, 14].	Standard protocols for stakeholder responsibilities and communication during stock-outs [14].
Independent pharmacies in the private sector need more time to acquire medicines. Delivery times vary depending on the location, size, and working capital of the private pharmacies [36, 52].	
Lack of accountability when contracted supplier is unable to supply [2].	
Lack of urgency felt in the supply chain and arduous communication; not all procurement personnel aware NEML medicines should always be stocked [2].	
	A national stockpile may be instituted to help prevent shortages of critical medicines [10].

	Explore international agreements for rapid access to international supplies if a shortage occurs [10].
	Mandatory early notification of impending shortages helps prevent stock-outs [53].
**Healthcare delivery**	
Physician turnover limits the ability to predict prescribing patterns and forecast [26].	Treatment guidelines could aid more efficient procurement [38].
Physician prescribing habits of originator products lead to lower availability of generic insulins and inhaling devices [6, 7, 29, 50].	
The centralized nature of public services such as mental health and diabetes has limited their availability in rural areas [13, 54].	Decentralization and task-shifting of services from referral hospitals to lower-level health facilities and integrating NCD services, including mental health, with HIV and MCH services at the primary care level [25, 55].
	Establishing medical consortia to enable shared bidding and procurement among differently tiered health facilities, promoting availability at lower levels [56].
Flexibility in prescription regulations for medical technicians and nurses is needed in case of stock-outs so medical treatment can be continued with alternative products [31].	
	Routine quality assurance activities are associated with higher availability [13, 27].
**Dispensing**	
Quality of cancer medicines may be compromised due to preparing and mixing without equipment or training [2].	
Acquiring insulin from online pharmacies is risky, given that storage and transportation conditions are poorly regulated [52].	
	Waste mitigation of antineoplastics through coordination of patient appointments [9, 10].
	Removal of compounding fees for pediatric formulations [57].
**Use(r)**	
Patient migration risks hospitals exceeding quantity projections [1].	

This is an overview of barriers to available medicines and proposed interventions in the literature, categorized according to the pharmaceutical supply chain. EML = Essential Medicines List; MCH = maternal and child health; NCD = non-communicable disease.

Barrier/recommendation implicates specific therapeutic area:

Respiratory diseases | Cancer | Diabetes | Mental Health

References

1. Moye-Holz D, Dreser A, van Dijk JP, Reijneveld SA, Hogerzeil HV. Access to cancer medication in public hospitals in a middle-income country: The view of stakeholder. Res Social Adm Pharm. 2020 Sep;16(9):1255-63.

2. Joosse IR, van den Ham HA, Mantel-Teeuwisse AK, Perumal-Pillay VA, Suleman F. Access to childhood cancer medicines in South Africa: a health systems analysis of barriers and enablers. J Pharm Policy Pract. 2024 Jul 12;17(1):2372033.

3. Boateng R, Petricca K, Tang B, Parikh S, SinQuee-Brown C, Alexis C, et al. Determinants of access to childhood cancer medicines: a comparative, mixed-methods analysis of four Caribbean countries. Lancet Glob Health. 2021 Sep;9(9):e1314-e24.

4. Shukar S, Yang C, Khan SA, Bhutta OA. Anti-cancer medicine shortages in an oncology tertiary hospital of Pakistan: A five-year retrospective study. J Oncol Pharm Pract. 2022 Jul 15:10781552221114278.

5. Saad MA, Masood SN, Al Bache N, Odhaib SA, Belkhadir J, Shegem N. Accessibility and availability of insulin: A survey by International Diabetes Federation-Middle East and North Africa Region (IDF-MENA) Member Associations. Journal Of Diabetology. 2022 Dec;13(5):73-80.

6. Özler G, Işik O. Prices, availability and affordability of selected essential medicines for chronic diseases in Türkiye. East Mediterr Health J. 2023 Nov;26(11):850-60.

7. Shukar S, Zahoor F, Omer S, Awan SE, Yang C, Fang Y. Experience of Pharmacists with Anti-Cancer Medicine Shortages in Pakistan: Results of a Qualitative Study. Int J Environ Res Public Health. 2022 Dec 6;19(23):16373.

8. Cao Z, Wang L, Ma R, Hu Y, Bao B, Liu X, et al. Access to essential and innovative anti-cancer medicines: a longitudinal study in Nanjing, China. BMC Health Serv Res. 2024 Jul 11;24(1):802.

9. Boateng R, Renner L, Petricca K, Gupta S, Denburg A. Health system determinants of access to essential medicines for children with cancer in Ghana. BMJ Glob Health. 2020 Sep;5(9):e002906.

10. Decamp M, Joffe S, Fernandez CV, Faden RR, Unguru Y; Working Group on Chemotherapy Drug Shortages in Pediatric Oncology. Chemotherapy drug shortages in pediatric oncology: a consensus statement. Pediatrics. 2014 Mar;133(3):e716-24.

11. Petricca K, Kambugu J, Githang'a J, Macharia WM, Njuguna F, McLigeyo A, et al. Access to essential cancer medicines for children: a comparative mixed-methods analysis of availability, price, and health-system determinants in east Africa. Lancet Oncol. 2023 May;24(5):563-76.

12. Economist Intelligence Unit. Cancer medicines shortages in Europe Policy: recommendations to prevent and manage shortages. London: The Economist; 2017.

13. Hakim S, Chowdhury MAB, Ahmed NU, Uddin MJ. The availability of essential medicines for diabetes at health facilities in Bangladesh: evidence from 2014 and 2017 national surveys. BMC Health Serv Res. 2022 Mar;22(1):377.

14. Tang B, Bodkyn C, Gupta S, Denburg A. Access to WHO Essential Medicines for Childhood Cancer Care in Trinidad and Tobago: A Health System Analysis of Barriers and Enablers. JCO Glob Oncol. 2020;6:67-79.

15. Mbonyinshuti F, Takarinda KC, Ade S, Manzi M, Iradukunda PG, Kabatende J, et al. Evaluating the availability of essential drugs for hypertension, diabetes and asthma in rural Rwanda, 2018. Public Health Action. 2021 Mar;11(1):5-11.

16. Armstrong-Hough M, Sharma S, Kishore SP, Akiteng AR, Schwartz JI. Variation in the availability and cost of essential medicines for non-communicable diseases in Uganda: A descriptive time series analysis. PLoS One. 2020 Dec;15(12):e0241555.

17. Satheesh G, Sharma A, Puthean S, Ansil TPM, Jereena E, Raj Mishra S, et al. Availability, price and affordability of essential medicines for managing cardiovascular diseases and diabetes: a statewide survey in Kerala, India. Trop Med Int Health. 2020 Dec;25(12):1467-79.

18. Asare B. Price and availability of medicines for non-communicable diseases in Ghana. Accra: Republic of Ghana Ministry of Health; 2024.

19. Cortes J, Perez-García JM, Llombart-Cussac A, Curigliano G, El Saghir NS, Cardoso F, et al. Enhancing

global access to cancer medicines. CA Cancer J Clin. 2020 Mar;70(2):105-24.

20. Kokabisaghi F, Hashemi-Meshkini A, Obewal A, Ghavami V, Javan-Noughabi J, Shabanikiya H, et al. Availability and affordability of cardiovascular medicines in a major city of Afghanistan in 2020. DARU J Pharm Sci. 2022 Dec;30(2):343-50.

21. Kadam AB, Maigetter K, Jeffery R, Mistry NF, Weiss MG, Pollock AM. Correcting India's Chronic Shortage of Drug Inspectors to Ensure the Production and Distribution of Safte, High-Quality Medicines. Int J Health Policy Manag. 2016 Sep 1;5(9):535-542.

22. Petricca K, Carson L, Kambugu J, Denburg A. Strengthening access to cancer medicines for children in East Africa: policy options to enhance medicine procurement, forecasting, and regulations. Glob Health Res Policy. 2024 Jul 1;9(1):24.

23. Ramadaniati HU, Anggriani Y, Lepeska M, Beran D, Ewen M. Availability, price and affordability of insulin, delivery devices and self-monitoring blood glucose devices in Indonesia. PLoS One. 2024 Oct 3;19(10):e0309350.

24. Beran D, Zar HJ, Perrin C, Menezes AM, Burney P. Burden of asthma and chronic obstructive pulmonary disease and access to essential medicines in low-income and middle-income countries. Lancet Respir Med. 2015 Feb;3(2):159-170.

25. Barbui C, Dua T, Kolappa K, Saraceno B, Saxena S. Mapping actions to improve access to medicines for mental disorders in low and middle income countries. Epidemiol Psychiatr Sci. 2017 Oct;26(5):481-490.

26. Martei YM, Chiyapo S, Grover S, Ramogola-Masire D, Dryden-Peterson S, Shulman LN, et al. Availability of WHO Essential Medicines for Cancer Treatment in Botswana. J Glob Oncol. 2018 Sep;4:1-8.

27. Hakim S, Chowdhury MAB, Haque MA, Ahmed NU, Paul GK, Uddin MJ. The availability of essential medicines for cardiovascular diseases at healthcare facilities in low- and middle-income countries: The case of Bangladesh. PLOS Glob Public Health. 2022 Nov;2(11):e0001154.

28. Kibirige D, Atuhe D, Kampiire L, Kiggundu DS, Donggo P, Nabbaale J, et al. Access to medicines and diagnostic tests integral in the management of diabetes mellitus and cardiovascular diseases in Uganda: insights from the ACCODAD study. Int J Equity Health. 2017 Aug;16(1):154.

29. Ozoh OB, Eze JN, Garba BI, Ojo OO, Okorie EM, Yiltok E, et al. Nationwide survey of the availability and affordability of asthma and COPD medicines in Nigeria. Trop Med Int Health. 2021 Jan;26(1):54-65.

30. Babar ZUD, Lessing C, Mace C, Bissell K. The availability, pricing and affordability of three essential asthma medicines in 52 low- and middle-income countries. Pharmacoeconomics. 2013 Nov;31(11):1063-82.

31. Wagenaar BH, Stergachis A, Rao D, Hoek R, Cumbe V, Napúa M, et al. The availability of essential medicines for mental healthcare in Sofala, Mozambique. Glob Health Action. 2015 Jun 15;8:27942.

32. Mukundiyukuri JP, Irakiza JJ, Nyirahabimana N, Ng'ang'a L, Park PH, Ngoga G, et al. Availability, Costs and Stock-Outs of Essential NCD Drugs in Three Rural Rwandan Districts. Ann Glob Health. 2020 Sep;86(1):123.

33. Dabare PR, Wanigatunge CA, Beneragama BH. A national survey on availability, price and affordability of selected essential medicines for non communicable diseases in Sri Lanka. BMC Public Health. 2014 Aug 8;14:817.

34. Osuafor NG, Ukwe CV, Okonta M. Evaluation of availability, price, and affordability of cardiovascular, diabetes, and global medicines in Abuja, Nigeria. PLoS One. 2021 Aug;16(8):e0255567.

35. Eden T, Burns E, Freccero P, Renner L, Paintsil V, Dolendo M, et al. Are essential medicines available, reliable and affordable in low-middle income countries? J Cancer Policy. 2019 Mar;19(1):100180.

36. Kaiser AH, Hehman L, Forsberg BC, Simangolwa WM, Sundewall J. Availability, prices and affordability of essential medicines for treatment of diabetes and hypertension in private pharmacies in Zambia. PLoS One. 2019 Dec;14(12):e0226169.

37. van Mourik MS, Cameron A, Ewen M, Laing RO. Availability, price and affordability of cardiovascular medicines: a comparison across 36 countries using WHO/HAI data. BMC Cardiovasc Disord. 2010 Jun 9;10:25.

38. Higuchi M. Access to diabetes care and medicines in the Philippines. Asia Pac J Public Health. 2010 Jul;22(3 Suppl):96s-102s.

39. Faruqui N, Martiniuk A, Sharma A, Sharma C, Rathore B, Arora RS, et al. Evaluating access to essential medicines for treating childhood cancers: a medicines

availability, price and affordability study in New Delhi, India. BMJ Glob Health. 2019 Apr 23;4(2):e001379.

40. Alemu BA, Hailemariam FH. Price, Availability and Affordability of Anti-Cancer Medicines in Addis Ababa, Ethiopia. Risk Manag Healthc Policy. 2022 Dec 29;15:2421-2433.

41. Mattila PO, Biritwum RB, Babar ZUD. A comprehensive survey of cancer medicines prices, availability and affordability in Ghana. PLOS ONE. 2023 May;18(5).

42. Cardenas MK, Miranda JJ, Beran D. Delivery of Type 2 diabetes care in low- and middle-income countries: lessons from Lima, Peru. Diabet Med. 2016 Jun;33(6):752-60.

43. Tenorio-Mucha J, Lazo-Porras M, Hidalgo-Padilla L, Beran D, Ewen M. Insulin: prices, availability, and affordability in public and private Peruvian pharmacies. Rev Panam Salud Publica. 2019 Oct;43:e85.

44. Sharma A, Bhandari PM, Neupane D, Kaplan WA, Mishra SR. Challenges constraining insulin access in Nepal-a country with no local insulin production. Int Health. 2018 May;10(3):182-190.

45. Joosse IR, van den Ham HA, Mantel-Teeuwisse AK, Suleman F. Alignment in the registration, selection, procurement and reimbursement of essential medicines for childhood cancers in South Africa. BMJ Glob Health. 2023 Sep;8(9):e012309.

46. Wirtz VJ, Kaplan WA, Kwan GF, Laing RO. Access to Medications for Cardiovascular Diseases in Low- and Middle-Income Countries. Circulation. 2016 May;133(21):2076-85.

47. Beran D, Ewen M, Lipska K, Hirsch IB, Yudkin JS. Availability and Affordability of Essential Medicines: Implications for Global Diabetes Treatment. Curr Diab Rep. 2018 Jun;18(8):48.

48. Beran D, Abdraimova A, Akkazieva B, McKee M, Balabanova D, Yudkin JS. Diabetes in Kyrgyzstan: changes between 2002 and 2009. Int J Health Plann Manage. 2013 Apr-Jun;28(2):e121-37.

49. Odhaib S, Masood S, Shegem N, Khalifa S, Saad MA, Eltom M, et al. The status of insulin access in Middle East-North Africa region. J Diabetology. 2022 Dec;13(5):48-61.

50. Tenorio-Mucha J, Lazo-Porras M, Zafra J, Ewen M, Beran D. Using government data to understand the use and availability of medicines for hypertension and diabetes: lessons from Peru. J Pharm Policy Pract. 2022 Nov;15(1):86.

51. Elias MA, Pati MK, Aivalli P, Srinath B, Munegowda C, Shroff ZC, et al. Preparedness for delivering non-communicable disease services in primary care: access to medicines for diabetes and hypertension in a district in south India. BMJ Glob Health. 2018 Jan;2(Suppl 3):e000519.

52. Satheesh G, Unnikrishnan MK, Sharma A. Challenges constraining availability and affordability of insulin in Bengaluru region (Karnataka, India): evidence from a mixed-methods study. J Pharm Policy Pract. 2019 Oct;12:31.

53. Nonzee NJ, Luu TH. The Drug Shortage Crisis in the United States: Impact on Cancer Pharmaceutical Safety. Cancer Treat Res. 2019;171:75-92.

54. Rahman MA, Babaye Y, Bhat A, Collins PY, Kemp CG. Availability of two essential medicines for mental health in Bangladesh, the Democratic Republic of Congo, Haiti, Nepal, Malawi, Senegal, and Tanzania: Evidence from nationally representative samples of 7958 health facilities. J Glob Health. 2022 Aug;12:04063.

55. Gupta N, Coates MM, Bekele A, Dupuy R, Fénelon DL, Gage AD, et al. Availability of equipment and medications for non-communicable diseases and injuries at public first-referral level hospitals: a cross-sectional analysis of service provision assessments in eight low-income countries. BMJ Open. 2020 Oct;10(10):e038842.

56. Li W, Guo W, Chen H, Lu W, Yu S, Wang M, et al. Access to single-inhaler triple medicines for chronic obstructive pulmonary disease in China: a national survey on accessibility and utilisation. J Pharm Policy Pract. 2025 Mar 10;18(1):2466215.

57. Orubu ESF, Robert FO, Samuel M, Megbule D. Access to essential cardiovascular medicines for children: a pilot study of availability, price and affordability in Nigeria. Health Policy Plan. 2019 Dec 1;34(Supplement_3):iii20-iii26.
